# Biomaterial and Hydrogel Strategies for Regenerative Microenvironment Reconstruction in Peripheral Nerve Conduits

**DOI:** 10.3390/gels11110898

**Published:** 2025-11-09

**Authors:** Wenjing Zhang, Yang Zhang, Hailin Ma, Lingxin Duan, Wenxiang Zhang, Ling Ding, Yuhui Kou, Baoguo Jiang

**Affiliations:** 1 Shenzhen Clinical Research Center for Trauma Treatment, Shenzhen University General Hospital, Shenzhen University, Shenzhen 518055, China; 2 Guangdong Key Laboratory for Biomedical Measurements and Ultrasound Imaging, National-Regional Key Technology Engineering Laboratory for Medical Ultrasound, School of Biomedical Engineering, Faculty of Medicine, Shenzhen University, Shenzhen 518060, China; 3 National Center for Trauma Medicine, Beijing 100044, China; 4 Department of Orthopedics and Trauma, Peking University People’s Hospital, Beijing 100044, China

**Keywords:** peripheral nerve injury, nerve guidance conduits, regenerative microenvironment, biomaterials, hydrogels

## Abstract

Peripheral nerve injury (PNI) poses a major clinical challenge, frequently resulting in chronic pain, muscle atrophy, and long-term functional impairment. While autologous nerve grafting remains the gold standard for repairing long-gap defects, its application is limited by donor-site morbidity and limited tissue availability. Nerve guidance conduits (NGCs) have emerged as promising alternatives; however, their efficacy remains suboptimal, primarily because most fail to recapitulate the spatiotemporally coordinated regenerative microenvironment required for robust axonal extension, timely remyelination, and durable neurovascular integration. Key limitations of current designs include an inability to balance the bioactivity of natural materials with the tunability of synthetic polymers, insufficient nutrient and oxygen delivery for long-gap repair, and a lack of dynamic, stage-specific regulation of the healing process. Consequently, microenvironment reconstruction represents the central bottleneck to achieving effective regeneration. This review synthesizes recent advances in purposefully rebuilding the NGC microenvironment across three interdependent dimensions: (i) activation and functional regulation of Schwann cells; (ii) immunomodulation to resolve inflammation while promoting repair; (iii) angiogenesis to ensure metabolic support. We place special emphasis on biomaterial strategies, particularly advanced hydrogels that integrate physical, biochemical, and dynamic cues for spatiotemporally programmed regeneration. Finally, we outline design principles and translational considerations for next-generation NGCs aimed at closing the efficacy gap with autografts.

## 1. Introduction

Peripheral nerve injury (PNI) results in permanent limb dysfunction, manifested by chronic pain, muscle atrophy, and the partial or complete loss of motor and sensory functions [1,2]. Its profound impact on patients, their families, and society underscores the critical need to explore novel mechanisms of nerve regeneration and develop effective repair strategies. Consequently, improving the outcomes of PNI repair and reducing disability rates have become a major focus in trauma research, holding substantial clinical and scientific value [3]. The approach to repairing PNI varies depending on the injury type [4,5]. For injuries without substantial tissue loss, direct epineurial suturing or fascicular repair is typically performed. In cases of medium- to large-segment nerve defects, autografts or artificial nerve grafts are commonly used, while proximal nerve avulsions (e.g., brachial plexus injuries) often require nerve transfer or functional reconstruction techniques. Although microsurgery, neuroregenerative drugs, and functional rehabilitation have improved clinical outcomes, treating severe nerve root avulsions and large nerve defects remains challenging [6]. These complex cases continue to pose significant obstacles to satisfactory functional recovery, representing a major clinical bottleneck.

The repair of PNI is a complex process, often involving not only axonal damage but also injury to surrounding blood vessels, muscles, and connective tissues [2,7]. In such complex scenarios, traditional suturing or grafting techniques frequently yield limited success, resulting in repair failure or incomplete functional recovery [8]. Although peripheral nerves possess an intrinsic capacity for self-regeneration, this is often insufficient for complete functional restoration. When a large nerve gap forms, or when there is a significant diameter discrepancy between nerve ends during nerve transfer, grafts or conduits become necessary to bridge the stumps and support axonal growth [9,10]. In clinical practice, autologous nerve grafting remains the gold standard for long-gap PNI repair, as it provides a natural scaffold endowed with native Schwann cells and extracellular matrix. However, autografts are hampered by donor site morbidity, limited tissue availability, and issues of size mismatch, all of which can lead to suboptimal recovery [11]. To overcome these drawbacks, nerve guidance conduits (NGCs) have been developed as alternatives, providing a structured, biomaterial-based pathway to guide axonal regrowth [12].

Nerve conduits, as biomedical devices for repairing peripheral nerve defects, serve multiple crucial roles. Primarily, they shield the nerve stumps from the invasion of fibrotic tissues, while their internal microstructures (e.g., microgrooves and nanofibers) provide topographical guidance, promoting the orderly regeneration of nerves [13]. Furthermore, they can be functionalized with bioactive factors (e.g., nerve growth factor, NGF, and vascular endothelial growth factor, VEGF) or cells (e.g., Schwann cells) to optimize the regenerative microenvironment, thereby enhancing axonal regeneration and functional recovery [14,15]. Compared to traditional autografts, nerve conduits eliminate the donor-site morbidity and offer a minimally invasive and efficient alternative. Their design can also be personalized to meet the requirements of different nerve injuries, enabling the repair of long-segment defects and complex injuries. Our research group has applied nerve conduits in a small-gap sleeve-suture technique, which provides mechanical protection and creates a stable microenvironment conducive to the orderly growth of nerve fibers [7]. The combination of technique with nerve conduits provides a minimally invasive and efficient strategy for peripheral nerve repair with promising clinical application prospects. However, the effectiveness of nerve conduits in repairing long-segment defects, particularly those exceeding 3 cm, remains limited. This is likely due to insufficient nutrient supply within the conduit and the excessive distance for axonal growth. Achieving dynamic regulation of the regenerative microenvironment (e.g., using electroactive or responsive materials) and further optimizing personalized designs for different injury types remain significant challenges in the field [16].

The reconstruction of the biochemical–biophysical microenvironment is the central bottleneck in PNI repair, as it ultimately determines the success of regeneration within a conduit [17]. Effective recovery requires coordinated Schwann cell activation and maintenance of a repair phenotype, timely immune resolution to prevent chronic inflammation, and sufficient neovascularization to support axon elongation and myelination [18]. Biomaterials form the foundation for constructing medical devices like NGC implants designed to bridge nerve defects. Among them, hydrogels are a particularly versatile subclass; their hydrated, polymer network structure closely mimics neural tissue. However, current NGCs face three persistent limitations: (1) material trade-offs—natural matrices (e.g., collagen, chitosan) offer bioactivity but lack tunable strength and degradation profiles, whereas synthetics (e.g., PLGA, PCL) are tunable but bioinert without functionalization; (2) long-gap metabolic insufficiency, where inadequate nutrient and oxygen delivery across the conduit compromises repair; (3) a lack of dynamic, stage-specific control (e.g., electroactive or responsive systems) to match evolving repair needs. These constraints underscore why microenvironment reconstruction must be a primary design goal for next-generation NGCs.

In summary, while NGCs are promising alternatives to autografts, their efficacy in critical-sized defects remains limited by an inability to recapitulate the dynamic regenerative microenvironment. This review comprehensively analyzes current strategies for reconstructing this niche within NGCs. It begins by elucidating the pathophysiology of PNI, then examines interventions targeting three interdependent pillars: Schwann cell activation, immunomodulation, and angiogenesis. Finally, it synthesizes fundamental biological principles and advanced biomaterial toolkits to guide next-generation conduit design, thereby bridging basic research and translational application.

## 2. Pathophysiology of Peripheral Nerve Injury

The pathophysiology of peripheral nerve injury (PNI) follows a sequential, biphasic process, beginning with degeneration and followed by attempted regeneration. Immediately after trauma, the injured axon undergoes a cascade of degenerative events. At the injury site, the axonal membrane is disrupted, cytoskeletal components disintegrate, and ionic homeostasis is lost, leading to calcium influx and activation of calpains, which further propagate axonal breakdown [19]. The local damage triggers Wallerian degeneration in the distal nerve stump, where the separated axon fragments and the surrounding myelin sheath, which is essential for rapid signal conduction, begin to degenerate within 24–48 h [20]. Schwann cells are the most crucial glial cells in PNI repair and regeneration, playing a central role in Wallerian degeneration, axonal regeneration, and myelination [21,22,23,24]. Under normal physiological conditions, mature SCs differentiate into either myelinating SCs or non-myelinating SCs, which collectively provide structural and functional support to axons. Myelinating SCs form sheaths that tightly wrap around peripheral nerve processes to ensure efficient signal conduction. Following the loss of axonal contact, myelinating SCs dedifferentiate into a repair phenotype, downregulating myelin-related genes and ceasing myelin production [25]. These activated SCs proliferate and align into tubular structures called Bands of Büngner, providing physical guidance for regenerating axons. They also phagocytose myelin debris and secrete cytokines and neurotrophic factors such as NGF and glial cell line-derived neurotrophic factor (GDNF), which are crucial for neuronal survival and axonal regrowth [19,20]. Notably, activated SCs also become a primary source of potent angiogenic factors, most notably VEGF, initiating essential crosstalk with the vascular system [26]. The recruitment and activation of immune cells, particularly macrophages, represent another hallmark of this phase. SCs upregulate cytokines such as tumor necrosis factor-α, interleukin-1α, interleukin-1β (IL-1β), and monocyte chemoattractant protein-1, which attract macrophages [24]. These macrophages collaborate with SCs to clear residual axonal and myelin debris—a prerequisite for successful regeneration [27,28]. Initially, macrophages often exhibit a pro-inflammatory (M1) phenotype, which can exacerbate tissue damage through the release of reactive oxygen species (ROS) and pro-inflammatory cytokines like TNF-α and IL-1β if sustained. A timely transition to a pro-regenerative (M2) phenotype is critical to suppress inflammation and secrete factors that support SC function and tissue repair. Once the axons from proximal neurons successfully regenerate, repair phenotype SCs nearby redifferentiate into myelinating or non-myelinating SCs. New myelin sheaths then wrap the regenerated axons or form Remak bundles, re-establishing stable nerve signal conduction [29]. Therefore, promoting and sustaining the repair phenotype of SCs has emerged as a key therapeutic strategy for PNI repair [19,30].

**Figure 1:**
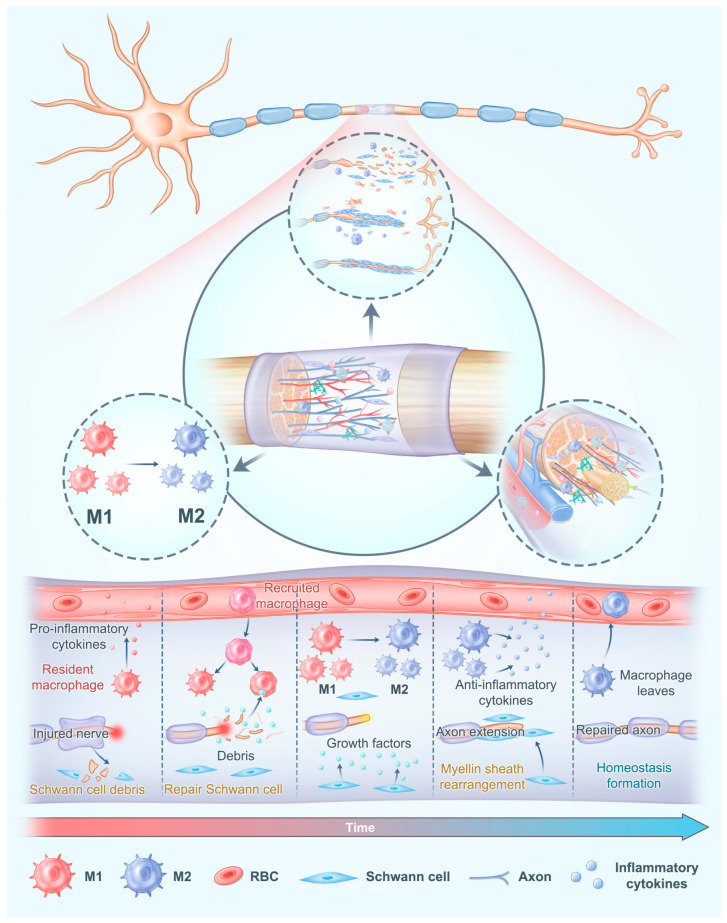
The regenerative microenvironment in nerve conduits requires comprehensive consideration of the activation and functional regulation of Schwann cells, precise modulation of the immune response, and sufficient regeneration of blood vessel. Adapted with permission from ref [34].

Despite this inherent regenerative capacity, functional recovery is often incomplete, particularly in long-gap injuries. Major challenges include the slow rate of axonal regeneration (~1 mm/day), misdirection of regenerating axons leading to faulty reinnervation, and the formation of fibrotic scar tissue that acts as a mechanical barrier [8,31]. Furthermore, in large nerve gaps, the prolonged absence of neurotrophic signals from the distal stump can induce neuronal apoptosis in the dorsal root ganglia and spinal cord. Concurrently, the extended ischemic period within the conduit core prior to vascular ingrowth creates a hostile microenvironment that compromises the survival of both SCs and regenerating axons [20,32]. This complex interplay of cellular responses and microenvironmental deficiencies underscores the limitations of the body’s innate repair mechanisms and highlights the need for therapeutic interventions, such as advanced nerve conduits, to steer these processes toward successful functional restoration. As illustrated in Figure 1, reconstructing an ideal regenerative microenvironment in nerve conduits requires comprehensive consideration of three key elements: Schwann cell activation and functional regulation, precise immune modulation, and supportive blood vessel regeneration [4,33].

## 3. Schwann Cell-Focused Strategies for Regenerative Microenvironment Reconstruction

Schwann cells (SCs) are pivotal “regeneration-supporting cells” in peripheral nerve repair [23]. Following injury, they transform into a specialized repair phenotype, known as Büngner cells, which orchestrate myelin debris clearance, secrete neurotrophic factors, and guide axonal regrowth. This reparative program is bolstered by the immune response, as SCs release cytokines to recruit macrophages that aid in myelin clearance and stimulate angiogenesis. However, the repair phenotype is intrinsically unstable and short-lived, a limitation now recognized as a major barrier to effective regeneration [31]. Its maintenance depends on molecular regulators such as the transcription factor c-Jun and NMDA receptor: the premature decline of this program often leads to failed regeneration [35]. This failure is characterized by SCs that fail to form proper guidance tracks, resulting in aberrant axonal pathfinding, and that undergo premature differentiation, leading to incomplete remyelination. Consequently, nerve conduits, by integrating physical, biochemical, and dynamic cues, are designed to comprehensively enhance SC-mediated regeneration, making them a critical component of modern nerve repair strategies.

### 3.1. Physical Support and Structural Guidance

**Figure 2:**
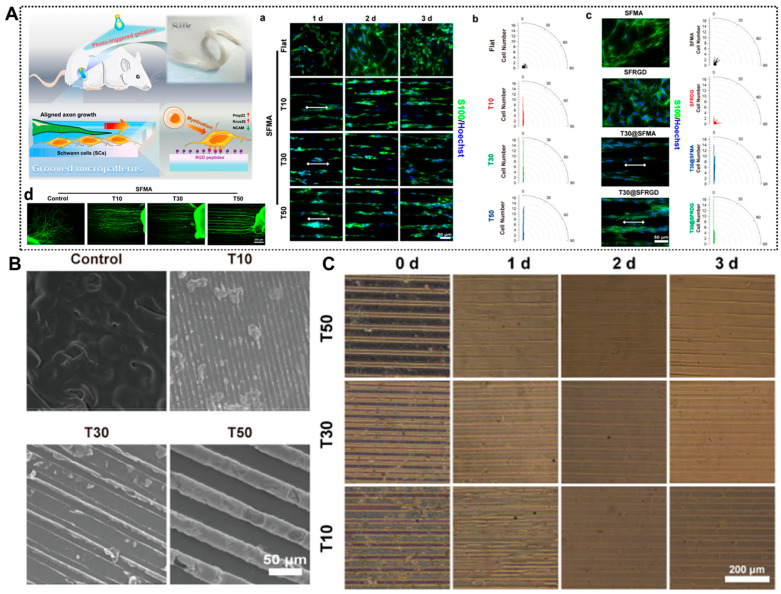
Bioengineered Structural Designs of Nerve Conduits with Physical Support and Structural Guidance. (**A**) Silk-based hydrogel conduits with a combination of therapeutic cues of topological micropatterns and RGD peptides to repair PNI via promoting rapid axon regeneration. (**a**) Aligned growth of SCs cultured on micropatterned hydrogels (T10, T30, and T50) characterized by immunofluorescence staining for SCs (S100, green) and the nucleus (Hoechst 33342, blue). (**b**) Orientation angle of SCs. (**c**) Fluorescence images and orientation angle of SCs cultured on hydrogels characterized by immunofluorescence staining for SCs and the nucleus after 3 days of incubation. (**d**) dorsal root ganglia (DRG) neurons (NF200, green) on micropatterned hydrogels characterized by immunofluorescence staining after 3 days of incubation. (**B**) SEM images of grooved micropatterns on hydrogels using PDMS molds (for the width of the channels, T10: 10 μm, T30: 30 μm, and T50: 50 μm). (**C**) Images of grooved micropatterns on the hydrogels after immersion in PBS. Adapted with permission from ref [36].

Nerve conduits provide a structured scaffold that physically supports Schwann cell alignment and migration, which is essential for forming “Bands of Büngner”—cellular structures that guide axonal growth. Micro-/nanopatterned conduits (e.g., grooves/ridges) align and elongate SCs along the injury axis, and microgrooved surfaces have been shown to accelerate SC migration and improve axonal alignment in vitro and in vivo. A silk-inspired phototriggered gelation nerve conduit, which combined anisotropic topological structures and adhesive ligands, improved the microenvironment for long-distance peripheral nerve regeneration [36]. The arg-gly-asp (RGD)-peptide-immobilized hydrogel scaffold promotes axonal growth, achieving cell recruitment of SCs and myelination. Meanwhile, the aligned groove micro-patterns promote the directional growth of SCs and the rapid growth of axons, providing significant nerve regeneration and functional recovery for long-distance nerve injuries (Figure 2). Zhou et al. [37] prepared a chitosan-based nerve conduit with longitudinal microchannels and nanofibers through a combination of unidirectional freezing and electrospinning. In vitro experiments showed that this uniquely structured nerve conduit significantly promotes the growth and migration of SCs and the elongation of neurites in PC-12 cells. This indicates that the synergistic effect of micro-and nano-structures can provide more effective physical support and guidance for SCs and neurites. Additionally, conduits with optimized pore sizes facilitate cell infiltration and nutrient diffusion, creating a more favorable environment for Schwann cell proliferation and migration [38].

### 3.2. Biochemical Modulation

Biochemical cues delivered by nerve conduits can markedly enhance SC activity. For example, NGF/BDNF/VEGF-loaded systems promote SC proliferation and migration; collagen conduits releasing NGF or GDNF maintain factor bioactivity for ≥30 days and enhance axonal regeneration [39]. Madduri et al. [40] prepared collagen nerve conduits (CNCs) that release GDNF and NGF through physical cross-linking and PLGA coating. These conduits can continuously release neurotrophic factors for more than 30 days, and the released factors maintain their biological activity. The study found that collagen-based nerve conduits containing NGF can significantly enhance the activity of SCs, thereby more effectively promoting axonal regeneration. Furthermore, the chemical modification of nerve conduits can enhance Schwann cell activity in multiple ways. Peptide modification, such as RGD (arginine–glycine–aspartate) [36], improves Schwann cell adhesion and promotes their activity. Recent studies have found that by constructing specific surface gradients, such as the concentration gradient of laminin-derived peptides, the migration of SCs can be effectively guided. The research of Motta et al. [41] found that the YIGSR peptide can not only promote the adhesion and proliferation of SCs but also guide their directional migration. Modifying the surface of nerve conduits with the YIGSR peptide can provide a more favorable microenvironment for SCs, promoting their secretion of neurotrophic factors and enhancing axonal regeneration ability.

### 3.3. Dynamic Cues and Electrical Stimulation

Electrical stimulation is a potent strategy for promoting peripheral nerve regeneration, and conductive materials have emerged as effective platforms for delivering such stimuli within nerve conduits. These materials, which include graphene, polypyrrole, and others, enhance Schwann cell proliferation and alignment through electroactive cues [42]. For instance, graphene oxide-coated conduits combined with low-frequency electrical stimulation have been shown to improve SC activity and myelination. In another example, Wu et al. [43] developed a highly tunable, conductive and biodegradable flexible polyurethane via the polycondensation of poly(glycerol sebacate) and aniline pentamer. This material significantly upregulated myelin gene expression and neurotrophic factor secretion in SCs. Culturing SCs on these conductive polymer membranes confirmed their biocompatibility and sustained ability to promote myelination and trophic support. A wide range of conductive materials—such as polymers (e.g., chiral hydrogel [44], zwitterionic hydrogel [45], polypyrrole [46,47,48]), metals (e.g., gold [49]), and carbon-based systems (e.g., graphene [50], graphdiyne [51], carbon nanotubes [52])—are gaining increasing attention in nerve conduit design. Owing to their unique combination of electrical conductivity and biocompatibility, these materials can effectively modulate Schwann cell proliferation, migration, and alignment, thereby facilitating functional nerve regeneration.

### 3.4. Cell Seeding and Function Enhancement

**Figure 3:**
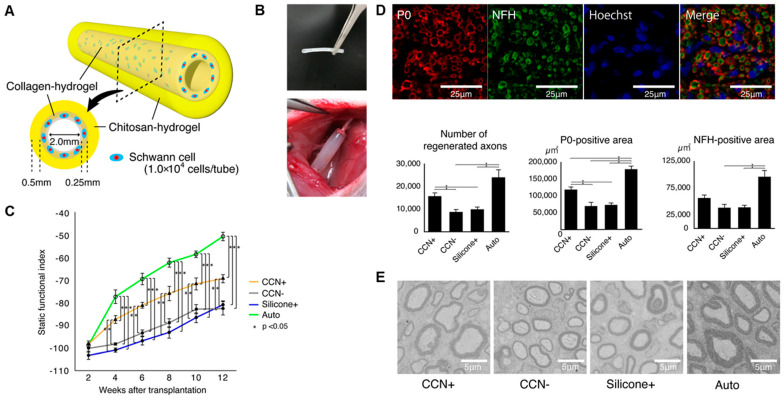
Schwann cell-encapsulated chitosan–collagen hydrogel nerve conduit promotes peripheral nerve regeneration in rodent sciatic nerve defect models. (**A**) The concept of our chitosan–collagen hydrogel nerve conduit (CCN). (**B**) Images of the CCN before transplantation and after transplantation to a sciatic nerve. (**C**) SFI for motor functional evaluation. (**D**) Fluorescence immunohistochemistry to assess axonal regrowth and myelination in the four groups. (**E**) Representative electron microscopic images in the central axial sections of all groups at 12 weeks after transplantation. Adapted with permission from ref [53].

Seeding Schwann cells or Schwann-like cells into nerve conduits provides direct cellular support to the regenerating nerve and is a major strategy to enhance repair efficiency at the cellular level. Takeya et al. [53] fabricated chitosan–collagen hydrogel nerve conduits (CCNs) encapsulated with SCs and transplanted them into a rat sciatic nerve defect model (Figure 3). Conduits seeded with SCs (CCN^+^) outperformed unseeded CCN (CCN^−^) and silicone tubes seeded with SCs in terms of motor function recovery, axonal regeneration, and remyelination. After 12 weeks, the CCN^+^ group showed a higher sciatic nerve function index (SFI), thicker myelin, and a greater number of regenerated axons, with stronger P0 (myelin) and NFH (axon) signals (Figure 3B). Although transplanted SCs were no longer detectable after 4 weeks, early paracrine secretion likely initiated a pro-regenerative environment that sustained repair. Beyond direct SC seeding, gene-modified SCs can be introduced to further boost trophic support. For instance, Schwann cells electrotransfected with plasmids encoding BDNF were seeded onto highly aligned PLA–PPy ultrafine fibers and co-cultured with dorsal root ganglia (DRG), leading to increased neurite extension and branching [54]. In this setting, the scaffold offered oriented physical guidance, while BDNF released by the modified SCs engaged TrkB receptors on DRG neurons to enhance survival and axonal outgrowth. These findings indicate that transiently delivered, pro-regenerative Schwann cell phenotypes, especially genetically enhanced ones, can leave a durable functional imprint on the regenerating nerve.

### 3.5. Inflammation Regulation and Environment Optimization

Nerve conduits can be functionalized to modulate the inflammatory response and create a more favorable microenvironment for SCs. Following nerve injury, excessive inflammation is often detrimental to SCs survival and function. Incorporating anti-inflammatory molecules such as IL-10 or TGF-β into conduits can effectively mitigate this response [55]. IL-10 inhibits the activation of inflammatory cells and the release of pro-inflammatory factors, thereby helping to maintain a stable environment for SCs. In contrast, TGF-β promotes ECM synthesis and cell proliferation, facilitating repair at the injury site. For instance, chitosan-based conduits releasing IL-10 have been shown to reduce inflammation significantly, directly promoting SCs proliferation and healthy axonal growth. Beyond inflammation, oxygen deficiency within the conduit presents another major barrier to SCs in the early stage of transplantation phase [26]. To address this, Ma et al. [56] developed a synthetic oxygen carrier (PFTBA)-enriched fibrin hydrogel, that provided sustained oxygen release for up to 96 h, markedly significantly improving SC survival, migration, and neurotrophic factor secretion under hypoxic conditions. These findings highlight the potential of oxygen-supplementing hydrogel systems as a transitional strategy prior to the establishment of neovascularization.

## 4. Immunomodulation in Nerve Conduits

### 4.1. Spatiotemporal Dynamics of the Immune Response After Peripheral Nerve Injury

A precisely orchestrated immune response unfolds following PNI, playing a pivotal yet double-edged role: it is essential for initiating repair but can impede regeneration if dysregulated. This process involves the sequential recruitment and activation of various immune cells, with macrophages serving as the central players. Supporting this, Büttner et al. [57] demonstrated that in age-associated nerve injuries, macrophage infiltration and inflammatory factors factor expression deviate from the typical pattern. This dysregulation diminishes nerve regeneration capacity, underscoring the critical role of macrophages in shaping the regeneration microenvironment.

#### 4.1.1. Acute Phase: Debris Clearance and Initiation of Repair

The inflammatory response is rapidly initiated following nerve injury, serving as an essential mechanism for self-protection and repair. This response involves the prompt recruitment of numerous immune cells, including macrophages and neutrophils, to the injury site [58,59]. Macrophages are pivotal this process; their high plasticity allows them to adopt different phenotypes in response to microenvironmental signals, primarily polarizing into the classically activated M1 or alternatively activated M2 [60]. In the early phase of nerve injury, macrophages predominantly assume the M1 phenotype. These cells exert potent pro-inflammatory fand bactericidal effects by secreting and bactericidal effects by secreting large quantities of cytokines such as TNF-α, IL-1β, and IL-6, and by generating cytotoxic substances including ROS and NO to eliminate pathogens and clear damaged tissue debris. For example, following sciatic nerve injury, rapidly accumulated M1 macrophages phagocytose necrotic tissue, preventing secondary toxicity and clearing the way for neural stem cell proliferation and differentiation [61]. However, when over-activated, sustained M1 activity can be detrimental. The continuous release of inflammatory mediators exacerbates the local inflammatory response, leading to collateral damage to nerve tissues. Moreover, during phagocytosis, macrophages secrete factors like transforming growth factor-β (TGF-β) and insulin-like growth factor (IGF), which promote fibroblast proliferation and migration. This, in turn, accelerates the synthesis and remodeling of the ECM and lays a structural foundation for subsequent nerve regeneration.

#### 4.1.2. Resolution Phase: Phenotype Transition and Tissue Remodeling

As the repair process advances, macrophages progressively polarize toward the M2 phenotype, known as alternatively activated macrophages. These M2 macrophages exert anti-inflammatory and tissue repair-promoting pro-regenerative functions, primarily through the secretion of cytokines IL-10, TGF-β, etc. These factors help to suppress inflammation, promote the synthesis and remodeling of the ECM, stimulate the proliferation and differentiation of neural stem cells, and facilitate axonal regeneration [60]. Consequently, in models of PNI, strategies that skew macrophage polarization toward the M2 phenotype can significantly improve regenerative outcomes. This is supported by studies showing that bioactive molecules like interleukin-4 (IL-4) can induce M2 polarization, thereby creating a microenvironment conducive to nerve regeneration [62]. Beyond macrophages, other immune cell types are integral to coordinating nerve repair [28]. Neutrophils, as the first responders, clear cellular debris and recruit monocytes via cytokines (e.g., IL-1β, TNF-α) and chemokines (e.g., CCL2), although their over-activation can be detrimental [63,64,65]. In later stages, T lymphocytes contribute in a subset-specific manner: while Th1 cells can exacerbate inflammation, Th2 cells secrete IL-4 and IL-10, thereby reinforcing M2 macrophage polarization and sustaining an anti-inflammatory milieu [66]. B lymphocytes also play a role, contributing through antibody production and the release of immunomodulatory cytokines like IL-6 and IL-10, though their precise functions require further elucidation [67].

#### 4.1.3. Dysregulation and Chronic Inflammation: A Barrier to Regeneration

While a moderate inflammatory response supports nerve regeneration, excessive or persistent inflammation. A chronic inflammatory state releases an abundance a large amount of pro-inflammatory factors that trigger the over-proliferation and activation of fibroblasts. This, in turn, stimulates the excessive synthesis of ECM components like collagen and fibronectin leading to scar tissue formation [61,66]. In f peripheral nerve repair, such scar tissue act as a physical barrier impeding axonal growth and preventing regenerating axons from crossing the lesion site. Moreover, excessive inflammation promotes the accumulation of neurotoxic substances, including ROS and NO, which directly damage neurons and SCs, inducing apoptosis and necrosis, thereby further suppressing regeneration.

This vicious cycle underscores the therapeutic potential of nerve conduits capable of modulating the immune response. By effectively regulating inflammation, conduits can avert these adverse effects and foster a favorable microenvironment for regeneration. Thus, immunomodulation is vital for creating a supportive repair niche. It acts synergistically with other repair mechanism. An anti-inflammatory milieu promotes SC proliferation and secretory function, facilitating myelination and axonal regrowth. Furthermore, immunomodulatory factors, like VEGF and TGF-β possess dual functions—they not only mitigate inflammation but also stimulate angiogenesis, thereby providing essential nutritional support and improving the local ECM balance for optimal repair [68]. Consequently, strategic immune modulation is indispensable for successfully optimizing the regenerative microenvironment.

### 4.2. Immunomodulation Strategies

Recent advances in immunomodulatory nerve conduits have significantly improved outcomes for PNI repair. The strategic manipulation of the local immune environment is now recognized as a critical factor for successful regeneration, and nerve conduits offer an ideal platform for this purpose. A primary focus is guiding the polarization of macrophages from the pro-inflammatory M1 phenotype toward the anti-inflammatory, pro-regenerative M2 state [69]. The development of biomaterials is central to this strategy. Nerve conduits serve as exceptional drug delivery systems, enabling the localized release of anti-inflammatory agents directly at the injury site. This approach maximizes therapeutic efficacy while minimizing systemic side effects. Furthermore, the intrinsic properties of the conduit material itself are pivotal. Hydrogels, for instance, can be incorporated as components or coatings, and their immunomodulatory potential can be fine-tuned by engineering physical and chemical parameters such as pore size, surface charge, and degradation rate to direct immune cell behavior. Natural biomaterials like collagen and chitosan provide good biocompatibility and support nerve cell adhesion. Their functionality can be further enhanced through modification with immunomodulatory molecules or cell adhesive peptides, creating conduits that actively promote a healing microenvironment. Several specific functionalization strategies are being actively explored:

Bioactive Molecules: Conduits are being functionalized with factors like IL-10 and TGF-β to directly stimulate M2 macrophage polarization. This shift enhances anti-inflammatory responses, promotes angiogenesis, and creates a microenvironment that supports Schwann cell activity and axonal regeneration [62].

Dual-Function Conduits: There is growing interest in conduits that simultaneously modulate immunity and enhance angiogenesis. For example, VEGF- or FGF-loaded conduits not only stimulate new blood vessel formation but also indirectly calibrate macrophage activity, ensuring a balanced immune response that supports—rather than hinders—axonal regeneration [70].

Cell-Based and Cell-Free Therapies: Seeding conduits with mesenchymal stem cells (MSCs) or SCs leverages their innate capacity to secrete immunomodulatory factors that reduce inflammation and promote repair [71]. A more recent and promising direction involves using exosomes derived from these cells [72]. These nanovesicles carry miRNAs and proteins that can directly modulate macrophage activity and promote axonal growth, offering a potent, cell-free strategy for immune regulation.

In summary, while the immune response plays a complex dual role in nerve repair, nerve conduits are indispensable tools for harnessing it beneficially. However, challenges persist, including achieving precise spatiotemporal control over immunomodulation, minimizing off-target effects, and ensuring long-term conduit stability [73]. A deeper understanding of immune mechanisms, coupled with the continued development of precisely engineered conduit-based is essential for advancing promoting peripheral nerve regeneration and functional recovery.

## 5. Angiogenesis in Nerve Conduits

### 5.1. The Significance of Angiogenesis in the Microenvironment for PNI Repair

**Figure 4:**
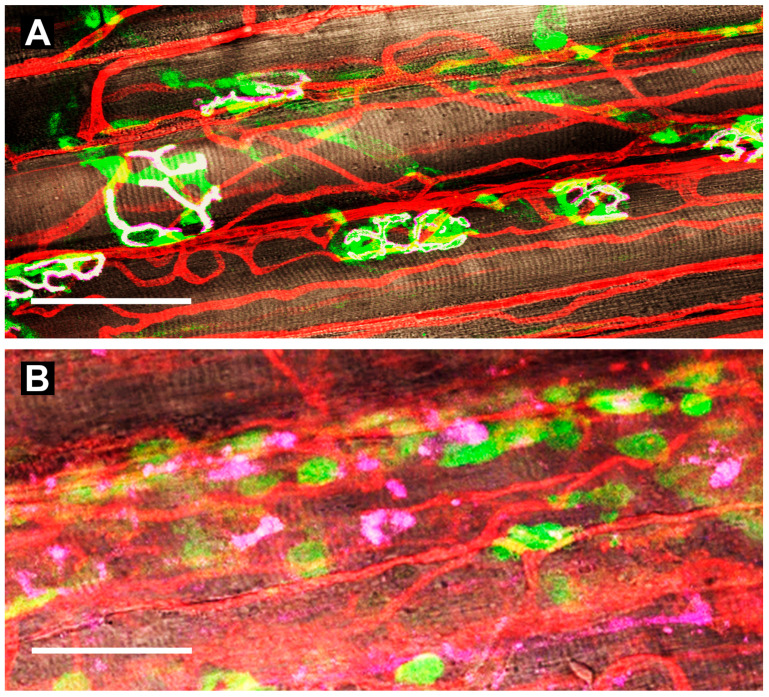
Neuromuscular junctions and capillaries in the gluteus maximus muscle of mice. (**A**) uninjured controls. Schwann cells [S100B-EGFP (green; 509 nm peak fluorescence)] enshroud axons as they terminate at the motor endplate above nicotinic acetylcholine receptors (AChRs) labeled with α-bungarotoxin 555 (pink) expressed on parallel myofibers (brown) with sarcomeres in register (white indicates overlap). Capillaries stained with wheat germ agglutinin 647 bound to their glycocalyx (red) transport oxygen and nutrients while removing metabolic by-products. (**B**) at 7 days post-injury (barium chloride injection), Schwann cells localize along regenerated capillaries (note proliferation and tortuosity); AChRs are fragmented and dispersed along regenerating myofibers devoid of innervation. Scale bars = 50 µm. Adapted with permission from ref [75].

Peripheral nerve regeneration is metabolically demanding, requiring a continuous supply of oxygen and nutrients for axonal elongation, remyelination, and matrix remodeling. These demands increase sharply after injury, while the core of a long-gap conduit often becomes hypoxic and nutrient-deprived. Therefore, timely and sufficient angiogenesis is a cornerstone of a pro-regenerative microenvironment and is tightly coupled to successful axon regrowth (Figure 4) [32,74,75]. Nerve conduits play a pivotal role in orchestrating regeneration, and their ability to promote vascularization is fundamental. They facilitate angiogenesis through two primary mechanisms. First, by acting as a physical guiding scaffold that directs the orderly migration and proliferation of vascular endothelial cells. Second, by serving as localized delivery platforms for pro-angiogenic factors such as VEGF. For instance, VEGF-loaded conduits can sustain the local release of this key cytokine, potently stimulating endothelial cell activity and driving neovascularization [76].

Within the conduit microenvironment, angiogenesis and neuroregeneration engage in a reciprocal dialogue. Activated SCs and other glia secrete VEGF and neurotrophin-3 (NT-3), which promote endothelial cell proliferation and migration. In turn, the newly formed blood vessels supply essential metabolic support and release factors such as IGF and TGF-β. These factors influence neural stem/progenitor cell fate, sustain SC activity, and guide axonal orientation [39,74]. Thus, the NGC functions not as a passive tube, but as a “bridge and amplifier” of neurovascular interactions. The spatiotemporal coordination of these processes is therefore paramount [77]. Temporally, the inflammatory response shortly after implantation helps initiate angiogenesis. Immune cells such as macrophages release inflammatory mediators that stimulate the expression of VEGF, setting the stage for vascular ingrowth [78]. As the vascular network matures, it ensures the sustained delivery of oxygen, nutrients, and ECM components, which is indispensable for subsequent phases of SC migration, axonal extension, and myelination. In the spatial dimension, the internal architecture of the conduit promotes the formation of stable “neurovascular units,” where regenerating nerve fibers and new capillaries are intimately associated [79]. This close physical alignment, often guided by micro-grooves or aligned fibers within the conduit, ensures efficient nutrient exchange. Simultaneously, the nerves provide trophic support to the nascent vasculature via secreted factors, creating a positive feedback loop that stabilizes the nascent microcirculation [78,80].

### 5.2. Key Mechanisms and Strategies to Promote Angiogenesis

The spatiotemporal coordination between blood vessels and nerves during regeneration is precisely regulated by a concert of factors within the nerve conduit microenvironment, including growth factors, cytokines, and the ECM. Among these, VEGF serves as a master regulator, directly promoting angiogenesis and influencing nerve regeneration by interacting with receptors on neuronal cells [74]. Similarly, neurotrophic factors like NGF and BDNF, while crucial for nerve regeneration, also exhibit pro-angiogenic properties. ECM components such as fibronectin and laminin provide essential physical support for both vascular endothelial cells and neurons. Furthermore, by engaging integrin receptors on cell surfaces, they activate intracellular signaling pathways that co-regulate angiogenesis and nerve regeneration [78]. The VEGF family, particularly the extensively studied VEGF-A, is central to this process [81,82]. VEGF-A drives vascular development and maturation has been implicated in protecting motor and sensory neurons [78]. The timely expression of VEGF-A is critical that its insufficiency during the initial repair stage phase can compromise Schwann cell-mediated nerve repair [79]. Following PNI, multiple cell types contribute to the VEGF pool within the conduit microenvironment: macrophages release VEGF during phagocytosis to promote vascularization and [60], dedifferentiating SCs secrete VEGF to facilitate their own migration and directly activate endothelial cells, and neurons produce VEGF for self-maintenance and local vascular regulation.

**Figure 5:**
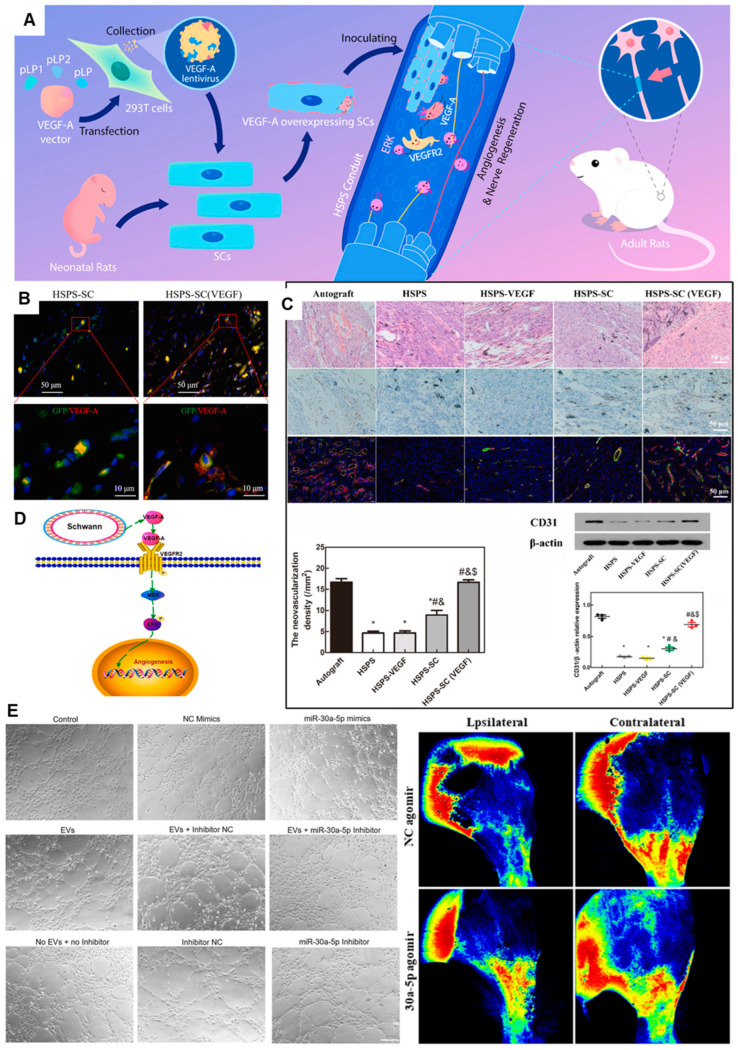
Conduit-Guided Neuroregeneration with VEGF-Overexpressing Schwann Cells Promotes Angiogenesis and Mechanistic Insights. (**A**) Comprehensive strategy of conduit guidance combined with VEGF producing SCs accelerates peripheral nerve repair. (**B**) Immunofluorescent co-staining of GFP and VEGF-A in vivo, green = GFP, red = VEGF-A, blue = DAPI. (**C**) Evaluation of angiogenesis in the regenerated nerve by H&E-staining, CD31 IHC-staining, CD34/α-SMA IF-staining and Western blot. *: *p* < 0.05, compared with the Autograft group; #: *p* < 0.05, compared with the HSPS group; &: *p* < 0.05, compared with the HSPS-VEGF group; $: *p* < 0.05, compared with the HSPS-SC group. (**D**) Possible mechanisms of VEGF-A overexpressing SCs to nerve regeneration. Adapted with permission from ref [81]. (**E**) miR-30a-5p exhibited proangiogenic effects both in vitro and in vivo. Adapted with permission from ref [84].

To harness these mechanisms, precise delivery of angiogenic factors is a cornerstone strategy. Gene delivery approaches utilize nerve conduits to localize viral (e.g., adenoviruses, lentiviruses) or non-viral (e.g., plasmid DNA, liposomes) vectors carrying the VEGF gene to the injury site. A notable example is the work of Wu et al. [81], who seeded VEGF-A-overexpressing SCs into a specialized conduit (Figure 5A–D). In immunofluorescence co-staining of GFP and VEGF-A, abundant VEGF-A could be detected both intracellularly and extracellularly in HSPS-SC(VEGF) group (Figure 5B). In a rat 10 mm-nerve-defect model, this strategy resulted in robust regeneration, with outcomes approaching those of autografts. Analysis revealed increased CD31+ endothelial cells and activation of the VEGFR2/ERK signaling pathway, underscoring the role of enhanced vascularization in the improved regeneration (Figure 5C–D). Protein-based delivery offers an alternative, focusing on the sustained release of recombinant factors like VEGF from carriers (e.g., microspheres, hydrogels) incorporated into the conduit. For instance, co-encapsulating VEGF and BDNF in AlgMA microsphere gels within a chitosan conduit has been shown to enhance the migration of Schwann cells (RSC96), reduce their apoptosis and stimulate tubulogenesis in human umbilical vein endothelial cells (HUVECs), thereby promoting a pro-regenerative environment [39].

Beyond single-factor delivery, cell-based and cell-free therapies show great promise. The conditioned medium from Induced pluripotent stem cell-derived mesenchymal stem cells (iMSC-CM) significantly enhanced angiogenesis in vitro. It robustly stimulated the migration of human umbilical vein endothelial cells (HUVECs) and their formation of tubular structures, concurrently increasing key quantitative metrics of vascular network complexity—including branch intervals, total branch length, and the number of nodes and meshes. It confirms that iMSCs promote angiogenesis through the secretion of pro-angiogenic cytokines [83]. Extending this concept, extracellular vesicles (EVs), particularly those derived from skin precursor-derived SCs (SKP-SC-EVs), have emerged as a potent, cell-free alternative. Shen et al. [84] demonstrated that SKP-SC-EVs significantly enhance angiogenesis and identified that miR-30a-5p as a key functional miRNA responsible for these effects both in vivo and in vitro (Figure 5E). The enrichment of miR-30a-5p in these EVs highlights its role as a critical angiogenesis regulator and opens a promising avenue for cell-free therapy in peripheral nerve repair.

## 6. Biological Principles and Engineering Strategies for Microenvironment Reconstruction

The strategic modulation of individual components—Schwann cell functionality, immune dynamics, and vascularization—as discussed in the preceding sections, represents a targeted yet fragmented approach to nerve repair within conduits. While these targeted interventions have significantly advanced the field, the inherent complexity and interdependence of the regeneration demand a more integrated paradigm. As research in materials, biology, and molecular signaling continues to expand and deepen, it becomes increasingly clear that successful microenvironment reconstruction must be grounded in a holistic understanding of the innate repair process. The following section distills this understanding into core principles that govern the design of next-generation conduits, ensuring that the collective cellular and molecular activities are spatiotemporally coordinated to mimic the natural cascade of nerve regeneration.

### 6.1. Biological Principles of Microenvironment Reconstruction

The primary objective of deploying NGCs transcends mere physical bridging of the nerve gap; it is to actively reconstruct a regenerative microenvironment that recapitulates key aspects of the natural healing process. This reconstruction is guided by several fundamental biological principles, aiming to orchestrate a conducive niche for the sequential events of nerve repair.

The first principle is the spatiotemporal orchestration of cellular responses. An ideal regenerative microenvironment must dynamically support the different phases of repair. In the acute phase, it should facilitate the clearance of debris via a controlled inflammatory response and support the activation and proliferation of SCs. In the intermediate phase, the focus shifts to guiding axonal elongation along aligned SCs and initiating angiogenesis to meet the escalating metabolic demands. In the final maturation phase, the environment must support axonal pruning, remyelination, and the formation of functional neuromuscular junctions [19,21,22,23,24]. Therefore, microenvironment reconstruction is not a static event but requires the temporal presentation of appropriate biological cues—such as early-release anti-inflammatory agents (e.g., IL-10) followed by sustained-release neurotrophic (e.g., NGF, BDNF) and angiogenic factors (e.g., VEGF)—to guide this complex sequence [27,59].

The second principle involves establishing a supportive biochemical and physical matrix. The extracellular matrix (ECM) within the conduit should mimic the endoneurial environment, providing not only a permissive substrate but also instructive signals. Biochemically, this entails the presentation of cell-adhesion peptides (e.g., RGD, IKVAV, YIGSR) to promote SC and neuronal adhesion, and the controlled delivery of growth factors to ensure cell survival, proliferation, and directed migration [36,39,85,86,87]. Physically, the matrix must provide topographical guidance (e.g., aligned nanofibers, microgrooves) to direct SC migration and axonal growth, a phenomenon known as “contact guidance” [13,36]. The mechanical properties of the conduit, such as stiffness and flexibility, should also match those of the native nerve to prevent fibrotic encapsulation and provide stable support without causing mechanical mismatch [18,88].

The third and most critical principle is fostering synergistic cellular crosstalk. The regenerative microenvironment is a communal platform where SCs, immune cells, and vascular endothelial cells interact in a tightly regulated network. SCs are the master regulators, secreting cytokines that recruit and polarize macrophages towards a pro-regenerative M2 phenotype, which in turn secretes factors that enhance SC migration and myelination [60]. Both SCs and M2 macrophages secrete VEGF and other angiogenic factors, promoting the formation of new blood vessels [73,78,80]. The nascent vasculature then reciprocates by supplying oxygen and nutrients, and endothelial cells themselves can secrete neurotrophic factors that support SC and neuronal health [75,79]. This creates a positive feedback loop—the “Schwann cells-immune cells-endothelial cells” axis—that is fundamental to efficient regeneration [20,32]. As demonstrated by Anne-Laure Cattin et al. [79], macrophages exhibit a unique sensitivity to the hypoxic environment within the nerve bridge following nerve injury. They secrete VEGF-A, which induces polarized vascular growth, thereby providing a guiding cue for Schwann cell migration. Reconstructing the microenvironment thus necessitates engineering strategies that potentiate this tricellular synergy, rather than addressing any single component in isolation.

### 6.2. Biomaterial Toolkits for Microenvironment Engineering

By serving as more than mere physical bridges, NGCs represent a cornerstone of peripheral nerve repair strategies. These advanced medical devices are designed to recapitulate critical elements of the native nerve microenvironment to guide and support regeneration. Their efficacy hinges on the biomaterials from which they are built—materials that provide structural integrity and can be functionalized with topographical cues (e.g., aligned microgrooves or nanofibers), complex internal architectures (e.g., multi-luminal channels), and advanced controlled-delivery systems for therapeutic agents [89]. To comprehensively regulate the regenerative microenvironment in PNI by nerve conduits, a series of synergistic regulation strategies can be adopted from multiple dimensions, including material selection, functionalization design, and construction of specific systems [90]. Ideal nerve conduit materials should possess excellent biocompatibility, biodegradability, and mechanical properties. The choice of biomaterial fundamentally dictates the conduit’s performance (Table 1). Natural biomaterials such as collagen and chitosan perform well in these aspects. Collagen, a major component of animal connective tissues and a key protein in the ECM, exhibits good biocompatibility, low immunogenicity, and hemostatic properties, providing natural adhesion sites for cells [88]. Chitosan, the only alkaline polysaccharide among natural polysaccharides, has a positive charge in an acidic environment due to its free amino groups. It possesses bioadhesion, antibacterial properties, and hemostatic effects, and can also promote axonal regeneration and prevent neuroma formation [91,92]. However, natural materials often suffer from batch-to-batch variability, inadequate and unpredictable mechanical strength, and degradation rates that are difficult to precisely control, particularly for long-gap repairs [93,94]. In contrast, synthetic polymers like poly(lactic-co-glycolic acid) (PLGA) and polycaprolactone (PCL) offer exceptional tunability. Their mechanical properties, degradation kinetics (from weeks to years), and geometry can be precisely engineered. PCL, in particular, is valued for its long-term stability and ease of processing into porous scaffolds favorable for cell infiltration. The primary drawback of synthetics is their general lack of bioactivity, often necessitating surface modification (e.g., RGD peptide conjugation) or blending with natural polymers to support specific cellular interactions [43].

**Figure 6:**
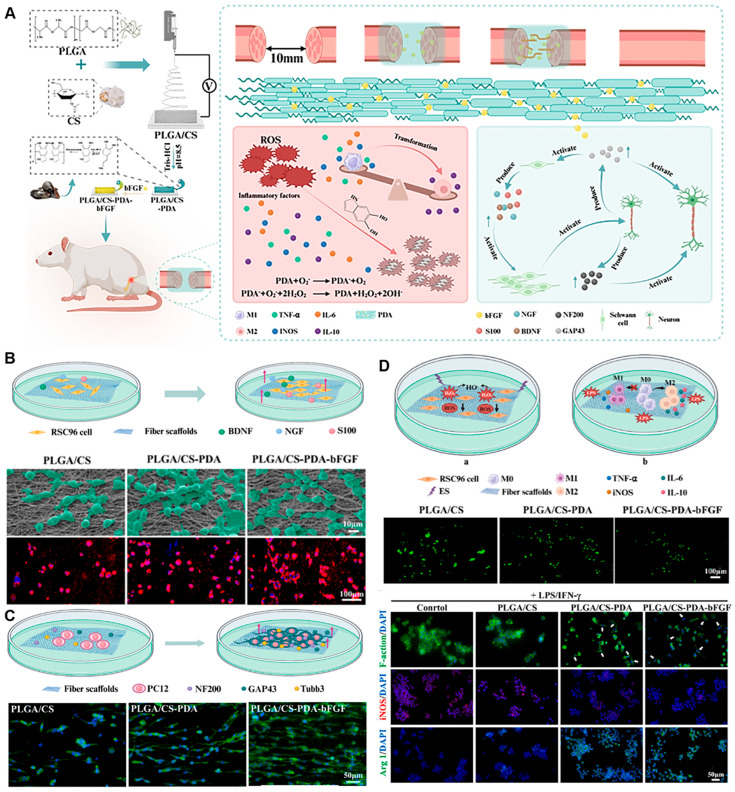
Immunomodulatory PLGA/Chitosan Aligned Nanofibers with Dopamine-Conjugated bFGF and ROS Scavenging Promote Peripheral Nerve Regeneration. (**A**) An immunoregulation PLGA/Chitosan aligned nanofibers with polydopamine coupling basic fibroblast growth factor and ROS scavenging for peripheral nerve regeneration. (**B**) SEM and CLSM images of SCs grown on scaffolds. Fluorescent indicator used was Alexa Fluor 594 for S100 (red), and the nuclei were stained with DAPI (blue). (**C**) The alignment of PC12 cells on scaffolds. (**D**) In vitro oxidative stress protection and immunomodulatory effects of scaffold groups. Adapted with permission from ref [117].

Hydrogels, as a versatile subset of biomaterials, have emerged as a pivotal platform for advanced NGC design [112]. These water-swollen, cross-linked polymer networks (e.g., based on gelatin methacryloyl (GelMA), hyaluronic acid, or alginate) uniquely mimic the hydrated, soft mechanics of native neural tissue. Their high permeability facilitates nutrient/waste diffusion, and their modular chemistry allows for precise incorporation of biochemical and biophysical cues. Hydrogels are exceptionally suited as intraluminal fillers to provide a permissive 3D matrix for cell migration and axonal growth, as coatings to functionalize conduit walls, or even as the primary conduit material for soft, compliant constructs. To systematically compare the properties and applications of various hydrogel-based nerve conduits, we summarize representative examples in Table 2, including their fabrication methods, advantages, limitations, and current research or clinical status [113,114,115]. Their capacity for the sustained and localized release of cells, exosomes, and bioactive factors (e.g., NGF, VEGF) makes them ideal for orchestrating the regenerative microenvironment [39,116]. A promising strategy involves creating composite materials that leverage the advantages of both natural and synthetic systems. For example, Xiaokun Chen et al. [117] fabricated an aligned nanofiber conduit from chitosan and PLGA, which was further functionalized with a polydopamine (PDA) coating. This composite not only provided superior mechanical guidance but also enabled the sustained release of bFGF, while the PDA coating contributed ROS-scavenging and immunomodulatory functions, leading to significant functional recovery in a rat sciatic nerve defect model (Figure 6).

### 6.3. Advanced Functionalization Strategies for Synergistic Regulation

#### 6.3.1. Electroactive and Topographical Guidance

**Figure 7:**
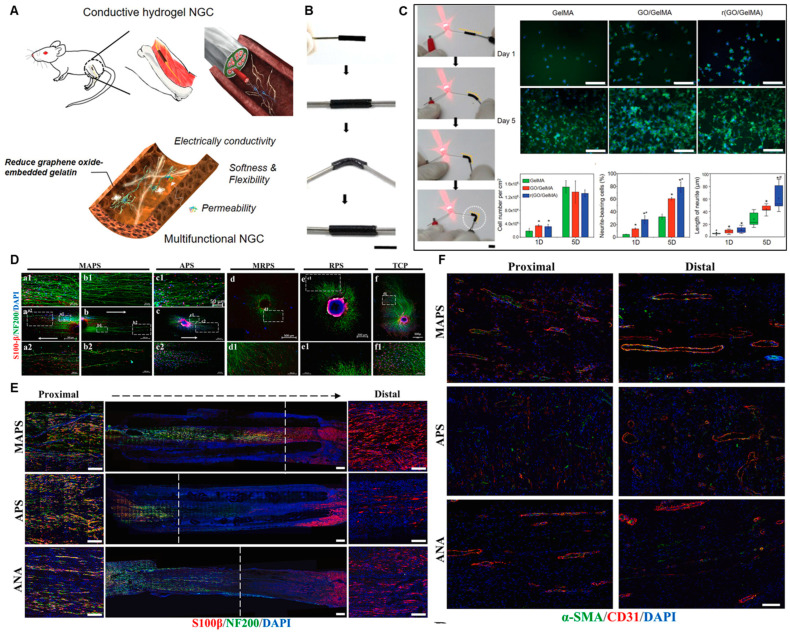
Multifunctional conductive scaffolds promote axonal growth and vascularization. (**A**) Schematic of a reduced GO/gelatin hydrogel-based nerve guidance conduit (NGC) with conductivity, flexibility, and permeability. (**B**) Photographs of a r(GO/GelMA) conduit during bending and connection with a cable and an LED bulb. (**C**) In vitro PC12 cell culture studies on various hydrogels. Scale bar: 100 µm. *, *p* < 0.05 compared to GelMA. #, *p* < 0.01 compared to GO/GelMA. Adapted with permission from ref [128]. (**D**) Immunofluorescence representative picture of DRG cultured on each group ((**a**–**f**) Schwann Cells: S100-β; Nerve axons: NF200; Nucleus: DAPI). (**E**) Representative images of IF staining of longitudinal section of graft (Schwann Cells: S100-β; Axons: NF200; Nucleus: DAPI) (Scale bar = 500 μm); The white dotted line indicates the length of regeneration from the Proximal end of the axon, and both sides display the Distal and Distal magnified renderings (Scale bar = 100 μm). (**F**) Vascular infiltration of regenerated nerve tissue inside the nerve catheter grafts in each group. α-smooth muscle actin: α-SMA; endothelial cells: CD31; Nucleus: DAPI (Scale bar = 100 μm). Groups: Acellular nerve allograft (ANA), purely aligned PCL/SF (APS), purely random PCL/SF (RPS), CDM-modified aligned PCL/SF (MAPS), and CDM-modified random PCL/SF (MRPS) nanofibers. Adapted with permission from ref [14].

Advanced nerve conduit design increasingly incorporates functional elements to actively direct regeneration. Key strategies include the integration of conductive components to mimic the endogenous bioelectrical environment, and the engineering of topographical and biochemical cues to guide cellular behaviors [127]. Integrating conductive materials into NGCs addresses the electrophysiological nature of nerve signaling, promoting Schwann cell (SC) proliferation, migration, and myelination. Beyond classic conductive polymers like polypyrrole (PPy), emerging carbon-based nanomaterials offer superior electrical and mechanical properties. As an emerging 2D carbon allotrope, graphdiyne (GDY) composites (e.g., with PCL) exhibit excellent conductivity and biocompatibility, significantly enhancing SC adhesion, proliferation, and glial gene expression in vitro [51]. When incorporated into hydrogels like GelMA, Graphene Oxide (GO) markedly enhances electrical conductivity (Figure 7A–C). The resulting r(GO/GelMA) hydrogel achieved a conductivity of 8.7 ± 1.6 mS cm^−1^, far exceeding pure GelMA (1.1 ± 0.1 mS cm^−1^), and robustly promoted neurite outgrowth from PC12 cells (Figure 7C) [128]. This highlights the advantage of carbon nanomaterials in creating electroactive scaffolds that support both glial and neuronal components. Physical architecture and surface chemistry are equally critical for directing cell migration and tissue integration. Aligned nanofibers with anisotropic topography provide contact guidance, directing the migration of SCs and promoting organized axonal regrowth. For instance, a double-biomimetic scaffold combining oriented PCL/silk fibroin nanofibers with a decellularized extracellular matrix (ECM) coating provided both physical alignment and biochemical signals, synergistically enhancing axon regeneration, SC migration, and angiogenesis in vivo [14] (Figure 7D-F). Coating conduits with functional molecules can further modulate the regenerative microenvironment. Polydopamine (PDA), a bio-adhesive polymer, has been used to modify chitin conduits, improving their mechanical properties, enhancing SC activity, and exerting anti-inflammatory effects that inhibit neuroma formation, achieving repair outcomes comparable to autografts in a 10 mm rat sciatic nerve defect [129]. In summary, the convergence of conductive composites (including emerging materials like graphene and GDY), instructive topographies, and sophisticated surface engineering provides a versatile toolkit for constructing next-generation, multifunctional nerve conduits. These strategies, often combined with stimuli-responsive (e.g., photo- or magneto-controlled) systems, enable the creation of dynamic, biomimetic microenvironments tailored for complex nerve regeneration.

#### 6.3.2. Spatiotemporal Delivery of Bioactive Cues

Achieving precise spatiotemporal control over the delivery of bioactive molecules is a cornerstone of advanced nerve conduit design. This strategy aims to replicate the natural sequence of nerve repair by providing the right signal at the right time and location. A leading approach involves multi-factor sustained-release systems that employ a hierarchical release strategy [130,131]. The conceptual ideal is the sequential release of distinct cues: early-stage anti-inflammatory cytokines (e.g., IL-10) to curb initial inflammation, mid-stage angiogenic factors (e.g., VEGF) to initiate vascularization, and late-stage neurotrophic factors (e.g., GDNF, BDNF) to support axonal maturation. Proof-of-concept for such temporal regulation is demonstrated by dual-factor systems. For instance, Zhang et al. [39] incorporated VEGF and BDNF into methacrylic alginate (AlgMA) microgels within chitosan conduits, which synergistically promoted the migration of rat Schwann cells (RSC96), reduced their apoptosis, and facilitated human umbilical vein endothelial cell (HUVEC) tube formation, thereby enhancing nerve regeneration. Beyond growth factors, the sustained release of small-molecule immunomodulators represents another powerful tool. Wang et al. [132] developed a chitosan-based semi-interpenetrating network hydrogel loaded with FK506. This scaffold acts as a “macrophage sponge,” recruiting macrophages and inducing their polarization to the pro-regenerative M2 phenotype via the upregulation of IL-10 and an increased CD206/TNF-α ratio. Concurrently, FK506 directly acts on Schwann cells, stimulating the secretion of myelin-related proteins (PMP22) and trophic factors (NGF, CNTF, VEGF), ultimately achieving myelin sheath reconstruction through multi-factorial synergy.

Complementing molecular delivery, ionic modulation has emerged as a potent and versatile strategy for microenvironment regulation. Among various ions, magnesium (Mg^2+^) has garnered significant attention due to its multi-faceted role in cellular processes [133,134,135,136]. Mg^2+^ exerts a concentration-dependent effect on neurite outgrowth, with optimal concentrations (10–40 mM) significantly promoting axonal extension in dorsal root ganglion neurons by activating the PI3K/Akt pathway and upregulating the axon guidance molecule Sema5b [137,138]. Furthermore, Mg^2+^ enhances the regenerative capacity of Schwann cells by activating the Wnt and CREB pathways, upregulating c-Jun expression, and driving them toward a repair phenotype that secretes elevated levels of NGF and BDNF [139]. Its immunomodulatory capability is evidenced by the suppression of the NF-κB pathway in M1 macrophages, reducing pro-inflammatory cytokines, while promoting an M2 phenotype [140]. This shift optimizes the regenerative milieu by reducing M1 infiltration and increasing M2 populations in vivo [137]. Additionally, Mg^2+^ promotes angiogenesis by activating the VEGF/VEGFR2 and HIF-1α pathways, directly stimulating endothelial cell proliferation and lumen formation [141,142]. The incorporation of Mg^2+^ into nerve conduits is achieved through material compounding (e.g., within chitosan, silk fibroin, or PCL) or surface modification, often using hydrogels or electrospun nanofiber membranes with layer-specific degradation kinetics to achieve sustained and spatiotemporally controlled release [137,138,139]. However, concentration-dependent effects of Mg^2+^ necessitate precise control, as levels exceeding 40 mM can inhibit axonal growth and concentrations above 2.0 mM may induce cytotoxicity [137]. The multi-dimensional regulatory potential of Mg^2+^ positions it as a key ion for developing innovative “bone-nerve cross-tissue repair materials,” with future work focused on optimizing its release kinetics and exploring synergistic combinations with other bioactive factors.

#### 6.3.3. Cell-Material Interaction Engineering

Cell-material interaction engineering has demonstrated immense potential in the field of PNI repair, primarily manifested in two key aspects. Firstly, establishing a co-culture system of SCs and endothelial cells holds significant promise. Research indicates that the interaction between these two cell types is beneficial for nerve regeneration. Zheng et al. [143] found that co-culturing SCs and endothelial cells on a chitosan/Artemisia sieversiana (CS/AS) scaffold with an anisotropic topological structure significantly improved the growth behavior of DRG. This system promotes DRG axon growth, provides stronger guidance for neuronal directional growth, prevents axon entanglement and knotting, stimulates the release of nerve growth factor and vascular endothelial growth factor, and regulates gene expression via relevant signaling pathways, thus creating a favorable microenvironment for nerve regeneration. Secondly, adopting genetically engineered cell delivery strategies offers multiple benefits. Implanting SCs overexpressing VEGF into nerve conduits can achieve neurotrophic support, immune regulation, and angiogenesis simultaneously. Huang et al. [82] developed a composite nerve conduit with an outer PLGA hollow tube and an inner layer of GelMA encapsulating VEGF-A transfected SCs. The VEGF-A overexpressing SCs stably secrete VEGF-A, promoting axon growth in DRG explants and neurons, and enhancing angiogenesis-related cell behaviors such as the migration and tube formation of human umbilical vein endothelial cells. In a rat sciatic nerve injury model, the nerve conduction and motor function recovery in the composite conduit group were comparable to those in the autologous transplantation group, validating the effectiveness of such genetically engineered cells in nerve repair. Additionally, another study showed that these cells could increase the recovery rate of nerve conduction velocity from 55% to 82%.

#### 6.3.4. Dynamic and Responsive Systems

**Figure 8:**
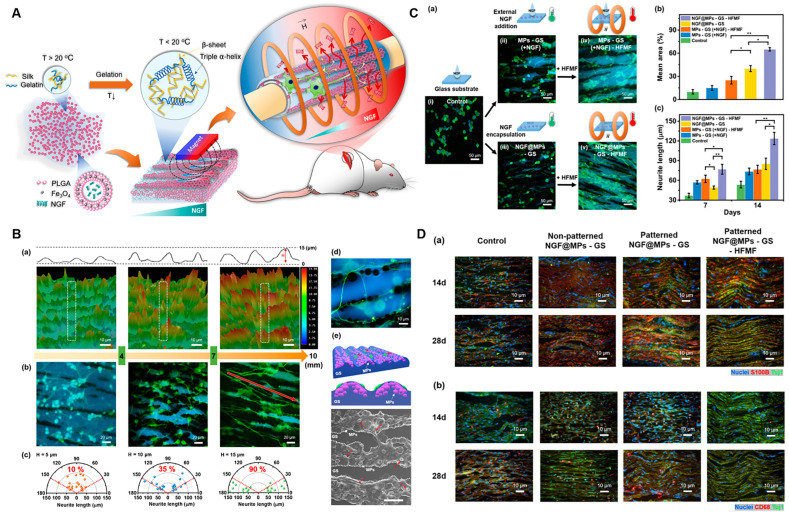
A magneto-responsive conduit with 3D-patterned NGF release promotes nerve regeneration. (**A**) Schematic Illustration of 3D Gradient and Linearly Aligned Magnetic Microcapsules in Nerve Guidance Conduits with Remotely Spatiotemporally Controlled Release to Enhance Peripheral Nerve Repair. (**B**) Topography of NGF@MPs-GS enables guidance of cell growth and differentiation. (**a**) Partition images of NGF@MPs-GS at *x*-axis positions of 3 mm, 6 mm, and 9 mm. Representative fluorescence images (**b**) and the polar graphs (**c**) showing statistic neurite length and orientations of cell differentiation on NGF@MPs-GS at *x*-axis positions of 3 mm, 6 mm, and 9 mm. (**d**) An enlarged fluorescence image revealing PC12 cells’ preferred growth and differentiation on MPs. (**e**) A schematic illustration and the corresponding SEM image showing that cell neurites can tightly interface with MPs to obtain NGF. (**C**) Cell Neurite Elongation on NGF@MPs-GS under a HFMF. (**a**) Representative fluorescence images of cell differentiation on (**i**) MPs-GS, (**ii**) MPs-GS with external NGF addition, called MPs-GS(+NGF), (**iii**) NGF@MPs-GS, (**iv**) MPs-GS–NGF followed by HFMF treatment, and (**v**) NGF@MPs-GS treated with HFMF. (**b**,**c**) the statistic cell density and neurite length, respectively, after 7 and 14 days of cell differentiation. * and ** indicated significant differences with *p* < 0.05 and *p* < 0.01, respectively, analyzed by one-way ANOVA with the post hoc Tukey HSD test (mean ± SEM). (**D**) Immunohistochemistry at wound sites of PNI after implantation. The experiment was conducted with four treatment groups: control, nonpatterned NGF@MPs-GS, patterned NGF@MPs-GS, and patterned NGF@MP-GS-HFMF at 4 weeks after implantation. (**a**) Immunohistochemistry staining with Schwann cell maker S100β and neuron marker Tuj1. (**b**) Immunohistochemistry staining with macrophage marker CD68 and neuron marker Tuj1. Adapted with permission from ref [145].

The development of dynamic, biomimetic materials that replicate the evolving microenvironment of natural nerve regeneration represents a critical frontier in nerve conduit research. Recent advances in material selection, structural design, and regulatory mechanisms, offering novel ideas and methods for more effective nerve repair. In terms of material and structural innovation, Song et al. [144] fabricated a shape-persistent conductive NGC by combining a thermoresponsive shape-memory polymer PLMC with gelatin, GO, or reduced graphene oxide (RGO) via electrospinning. The resulting multi-channel NGC features an outer layer of randomly arranged nanofibers and an inner layer of aligned nanofibers, mimicking the architecture of native nerve fascicles. The aligned fibers provide topological cues for axon elongation, while the thermoresponsive nature of the conduit ensures structural stability under physiological conditions, thereby maintaining a consistent physical microenvironment. Beyond structural and electrical cues, light and magneto-responsive materials offer unique advantages for spatiotemporal control over the regenerative process. A representative example is the work of Huang et al. [145] utilized the reversible thermoresponsive sol–gel phase transition ability of gelatin/silk fibroin (GS) hydrogel and combined it with magnetic PLGA microcapsules loaded with NGF (NGF@MPs) to construct a NGC named NGF@MPs-GS (Figure 8). As shown in Figure 8B–D, they verified that NGF@MPs could form 3D gradient-distributed and linearly arranged micropatterns in the GS hydrogel under magnetic guidance, which have a guiding effect on cell growth and differentiation. In both cell and animal experiments, it was confirmed that this NGC, with the assistance of a high-frequency magnetic field (HFMF), could control the spatiotemporal release of NGF, promote axon growth, myelin regeneration, and motor function recovery, and the implant did not trigger long-term or chronic inflammatory responses. This system provides a remotely controllable 3D gradient drug-delivery platform, offering a promising strategy for enhancing peripheral nerve regeneration. These intelligent systems can be triggered by external stimuli to achieve on-demand release of angiogenic factors, immunomodulators, and neurotrophic factors in a temporally defined sequence. Such dynamic regulation better mirrors the phased complexity of natural nerve repair—for instance, promoting angiogenesis and immunomodulation in early stages, and shifting toward neurotrophic support in later stages.

## 7. Conclusions and Future Perspectives

The evolution of NGCs has progressed from passive tubular scaffolds to bioactive, multifunctional systems designed to actively guide regeneration. Significant progress has been made in enhancing Schwann cell activation, orchestrating immunomodulation, and promoting angiogenesis, thereby enriching the design toolkit for NGCs. Nevertheless, clinical outcomes—especially for long-gap or complex injuries—continue to lag behind those of autografts. This gap stems from inherent limitations in current designs: most conduits cannot dynamically support the sequential phases of nerve repair, inadequately control early inflammation, fail to ensure sustained vascularization, and offer limited long-term support for remyelination. Practical challenges, including mechanical mismatch, unpredictable release kinetics of bioactive factors, batch-to-batch variability of natural materials, and constraints in sterilization and storage—further impede clinical translation.

Among biomaterial platforms, hydrogels are particularly promising due to their structural tunability, inherent biocompatibility, and capacity to integrate biochemical, physical, and dynamic cues. Future hydrogel-based NGCs should aim to deliver spatiotemporally programmed signals that couple immunomodulation with angiogenesis, replicate native electro-mechanical microenvironments to direct Schwann cell alignment and myelination, and employ ion-mediated regulation to guide axonal growth and macrophage polarization. The integration of living components (e.g., mesenchymal stem cells) or cell-free biologics (e.g., exosomes) may further enhance trophic support and synergize with structural and vascular regeneration cues.

Looking ahead, several key challenges and emerging directions must be addressed to advance the field:

A primary obstacle lies in achieving scalable, reproducible, and traceable GMP-compliant manufacturing of advanced biomaterials. For Class III devices, regulatory approval demands rigorous evidence of biocompatibility and sterilization compatibility (e.g., with ethylene oxide or gamma radiation) that preserves bioactivity without compromising mechanical integrity or degradation kinetics. Equally critical is establishing a robust long-term safety profile, which requires characterization of degradation by-products, local tissue responses, and potential neurotoxicity, supported by post-implantation follow-up extending beyond the acute phase of regeneration. Underpinning these efforts is the necessity for standardization; consistent control and reporting of properties such as degradation kinetics, mechanical strength, and bioactivity are indispensable for ensuring product reliability and facilitating successful regulatory review [113]. Addressing these translational hurdles is essential for the realistic clinical deployment of artificial NGCs across diverse indications.

There is an urgent need to establish uniform and clinically relevant in vivo models—particularly for long-gap nerve defects—that incorporate both neurophysiological and vascular regeneration endpoints. To enhance comparability across studies, we strongly recommend the adoption of standardized in vivo models alongside a minimum reporting set, which should explicitly include: species/strain, defect length, suture and fixation details, follow-up duration, and a core outcome panel comprising functional (e.g., SFI and/or gait analysis, CMAP), histological (axon counts, myelin thickness), and sensory assessments (e.g., 2PD where applicable). Furthermore, test conditions for mechanical characterization (tension/compression, dry vs. hydrated state, strain rate) and degradation assays (mass loss, degradation by-products) should be harmonized. Such standardization will enable meaningful head-to-head comparisons of different NGC designs, accelerate the identification of optimal regenerative strategies, and improve the translational relevance of preclinical data.

The economic viability and manufacturability of advanced NGCs—particularly those incorporating hydrogels, conductive polymers, or controlled-release systems—are critical determinants of their clinical adoption. Key considerations include production cost per conduit, process scalability (e.g., continuous versus batch production, yield), batch-to-batch consistency, and the expenses associated with sterilization and shelf-life management. Additionally, practical factors such as supply-chain robustness and GMP readiness serve as essential gatekeepers for multi-center deployment. To enhance translational potential, strategies such as modular design or simplified fabrication processes should be prioritized. Embedding these economic and operational constraints early in the development process will help align material selection and microenvironmental designs with real-world feasibility and broad accessibility.

Looking ahead, next-generation NGCs are poised to leverage emerging technologies such as 4D printing for dynamic, shape-adaptive conduits; magneto- or photo-responsive materials for remote spatiotemporal control; and bioelectronic interfaces for on-demand electrical stimulation. Furthermore, the integration of artificial intelligence in conduit design and personalized manufacturing promises to optimize performance based on patient-specific injury profiles. By addressing persistent translational challenges and harnessing these innovations, NGCs may evolve from static implants into dynamic, “stage-aware” systems that actively orchestrate regeneration. Ultimately, if these advanced platforms can successfully mediate coordinated crosstalk among glial, immune, and vascular cells, they hold the promise of narrowing the efficacy gap with autografts.

## Figures and Tables

**Table 1:** Quantitative comparison of commonly used nerve conduit materials.

Material	Mechanical Property	Degradation (In Vivo)	Gap & Model	Key Outcomes	Notes
Native tissue Peripheral nerve [95]	Young’s modulus ≈ 7 MPa (tension)				
Collagen (e.g., NeuraGen/NeuroFlex/NeuraWrap) [91,96,97,98,99]	Tensile strength: 0.41 ± 0.17 MPa (porous, unreinforced) to 3.69 ± 0.64 MPa (fiber-reinforced)	4–48 months (product-dependent; NeuroFlex ≈ 4–8 months; NeuraGen/Wrap up to ~48 months)	~12–17 mm	Gradual improvement in 2PD/sensory; for longer gaps often inferior to allograft/autograft	Values vary by porosity and reinforcement; report test mode/condition where available.
PGA (NeuroTube) [100,101]	NR (design uses corrugation/spiral to enhance kink resistance)	~3 months	Facial/digital nerve 20–25 mm	Acceptable recovery within a shorter support window	One of the earliest absorbable clinical conduits.
PCL [102,103,104]	Young’s modulus (tension): neat PCL ~204 ± 6.7 MPa; with PPy or similar ~35–51 MPa	~2–4 years (depends on Mw/crystallinity; maintains structure ≥ 18 weeks in vivo)	Rat sciatic 10–15 mm	Structurally stable; often needs bioactivity or compliance tuning	Compositing/blending can soften PCL and add cues (electrical/biochemical).
Chitosan (e.g., Nerbridge/custom CNC) [99,105,106,107]	Hydrated film: Young’s modulus ~30 ± 13 MPa; tensile strength ~47 ± 17 MPa; elongation at break ~101 ± 24%	~6–12 months or longer (tunable by acetylation/crosslinking)	Clinical multicenter digital nerve ≈ 17 mm; rat 10 mm common	For short gaps, functional outcomes approach autograft in some studies	Degree of deacetylation and crosslinking strongly affect both mechanics and degradation.
Silk fibroin (SF) conduits [108,109]	Tunable; commonly engineered to MPa-level tensile properties (comparable to nerve)	Months–years (depends on β-sheet content/crosslinking)	Rat sciatic 10–13 mm	Reports of SFI/CMAP comparable to autograft in selected models	Recent studies show good bridging at ~13 mm.
Gelatin hydrogels/composites [110]	Compressive/shear modulus (hydrated): ~20–57 kPa (increases with SF-MA/GO or other fillers)	Weeks–months (formulation-dependent)	Rat sciatic 10 mm common	Promotes Schwann cell support/axonal extension	Hydrogels emphasize soft-tissue matching; report compressive/viscoelastic metrics.
Hyaluronic acid (HA) hydrogel conduits [111]	NR (typically compressive/shear in kPa range; hydrated)	~6 months structure may persist (animal)	Rat short-gap models	Progressive tissue/functional improvement over time	Often used as soft, degradable sheath/filler around guidance structures.

**Table 2:** Hydrogel-based nerve conduits.

Material	Fabrication Method	Advantages	Disadvantages	Research/Clinical Status	Remarks
Fibrin hydrogel [118]	Fibrinogen + thrombin crosslinking	Excellent biocompatibility, supports axonal regeneration, can carry cells or growth factors	Low mechanical strength, rapid degradation, limited clinical data	15 mm sciatic nerve gap in rats	Often used as conduit filler
GelMA (Gelatin methacrylate) [119]	Gelatin methacrylation + photo-crosslinking	Tunable stiffness, photocurable tubular structures, supports axonal elongation	Limited light penetration, moderate mechanical strength	10 mm sciatic nerve defects in rats	Can be combined with microspheres or growth factors
Alginate hydrogel [120]	Sodium alginate + Ca^2+^ ionic crosslinking	Easy to prepare, injectable	Bio-inert, poor cell adhesion	1 cm sciatic defects in rats	Often combined with cells or growth factors
Decellularized ECM hydrogel (dECM) [121]	Decellularization → enzymatic digestion → re-gelation	Contains native neurotrophic cues, highly biomimetic	Batch variability, complex preparation	12 mm-gap sciatic nerve defect in rats	Can be customized from autologous/allogeneic sources
Collagen hydrogel [122]	Acidic collagen solution → molding → freeze-drying	Excellent biocompatibility, promotes Schwann cell adhesion and axonal growth	Low mechanical strength, rapid degradation	1cm gap in the rat sciatic nerve; FDA-approved, widely used clinically for short-gap peripheral nerve defects (<3 cm)	Well-established clinical evidence
Chitosan hydrogel [123]	Chitosan deacetylation → acid dissolution → casting/freeze-drying/crosslinking	Tunable degradation, antibacterial, good biocompatibility	Limited flexibility, low transparency	10 mm sciatic nerve defects in rats; early clinical evaluation	Suitable for short- to medium-gap defects
Silk fibroin hydrogel [50,124,125]	Silk fibroin extraction → degumming → freeze-drying/spinning/crosslinking	Good biocompatibility, high mechanical strength, can guide axonal growth	Complex fabrication, limited clinical data	10 mm defect of rat sciatic nerve; small-scale human exploratory studies	Aligned fiber structures enhance guidance
Synthetic hydrogels (PEG, PLGA-based) [126]	Solution casting/photo-crosslinking/electrospinning	Controllable degradation, tunable mechanical properties, can incorporate drugs or growth factors	Low inherent bioactivity, may produce acidic degradation products	Mostly preclinical/animal studies; limited clinical use	Often used for medium- to long-gap defects or as drug/cell delivery carriers

## Data Availability

No new data were created or analyzed in this study.
